# FEMORAL HERNIA: UNCOMMON, BUT ASSOCIATED WITH POTENTIALLY SEVERE COMPLICATIONS

**DOI:** 10.1590/0102-672020210002e1603

**Published:** 2021-10-18

**Authors:** Julio Cezar Uili COELHO, Faissal Nemer HAJAR, Gabriela Araujo MOREIRA, Andréa Virmond El HOSNI, Bruna Freitas SAENGER, Yan Sacha Hass AGUILERA, Marco Aurelio Raeder da COSTA, Christiano Marlo Paggi CLAUS

**Affiliations:** 1 Department of Surgery, Hospital Nossa Senhora das Graças, Curitiba, Paraná, Brazil

**Keywords:** Laparoscopic herniorrhaphy, Femoral hernia, Groin hernia, Totally extraperitoneal laparoscopy, Herniorrafia laparoscópica, Hérnia femoral, Hérnia na virilha, Laparoscopia totalmente extraperitoneal

## Abstract

**Background:**

Although the laparoscopic access is becoming the preferable treatment for femoral hernia, there are only few studies on this important subject.

**Aim:**

To assess the outcomes of the totally extraperitoneal laparoscopic (TEP) access in the treatment of femoral hernia.

**Methods:**

Data of 62 patients with femoral hernia who underwent herniorrhaphy were retrospectively reviewed. The diagnosis of femoral hernia was established by clinical and/or imaging exams in 55 patients and by laparoscopic findings in seven.

**Results:**

There were 55 (88.7%) females and 7 (11.3%) males, with female to male ratio of 8:1. The mean age was of 58.9±15.9 years, ranging from 22 to 92 years. Most patients (n=53; 85.5%) had single hernia and the remaining (n=9; 14.5%) bilateral, making a total of 71 hernias operated. Prior lower abdominal operations were recorded in 21 (33.9%) patients. Conversion to laparoscopic transabdominal preperitoneal procedure was performed in four (6.5%). Open herniorrhaphy was needed in two (3.2%), one with spontaneous enterocutaneous fistula in the groin region (Richter’s hernia) and the another with incidental perforation of the adjacent small bowel that occurred during dissection of hernia sac. There was no mortality.

**Conclusion:**

Femoral hernia is uncommon, and it may be associated with potentially severe complications. Most femoral hernias may be successfully treated with totally extraperitoneal laparoscopic access, with low conversion and complication rates.

## RESUMO

**Racional:**

Embora o acesso laparoscópico esteja se tornando o tratamento preferencial para a hérnia femoral, poucos são os estudos sobre esse importante assunto.

**Objetivo:**

Avaliar os resultados do acesso laparoscópico totalmente extraperitoneal no tratamento da hérnia femoral.

**Métodos:**

Os dados de 62 pacientes com hérnia femoral que foram submetidos a herniorrafia foram revisados ​​retrospectivamente. O diagnóstico foi estabelecido por exames clínicos e/ou de imagem em 55 pacientes e por achados laparoscópicos em sete.

**Resultados:**

Havia 55 (88,7%) mulheres e 7 (11,3%) homens, com proporção feminino/masculino de 8: 1. A média de idade foi de 58,9±15,9 anos (22-92). A maioria (n=53, 85,5%) apresentava hérnia única e o restante (n=9, 14,5%) bilaterais, perfazendo um total de 71 hérnias femorais operadas. Operações prévias no abdome inferior foram registradas em 21 (33,9%) pacientes. A conversão para procedimento pré-peritoneal transabdominal laparoscópico foi realizada em quatro (6,5%). Herniorrafia aberta foi necessária em dois pacientes (3,2%), um com fístula enterocutânea espontânea na região da virilha (hérnia de Richter) e o outro com perfuração incidental do intestino delgado adjacente que ocorreu durante a dissecção do saco herniário. Não houve mortalidade.

**Conclusão:**

A hérnia femoral é incomum e pode estar associada a complicações potencialmente graves. A maioria das hérnias femorais pode ser tratada com sucesso através do acesso laparoscópico totalmente extraperitoneal, com baixas taxas de conversão e complicações.

## INTRODUCTION

Femoral hernia is a protrusion of abdominopelvic content through the femoral ring into the femoral canal^9^ ^,^ ^18^. This canal is bordered by the inguinal ligament anterosuperiorly, Cooper’s ligament inferiorly, the femoral vein laterally and the junction of the iliopubic tract, and Cooper’s ligament (lacunar ligament) medially^18^. Because of the tendency of the femoral hernia to move upward to a position above the inguinal ligament, it may sometimes be mistaken for an inguinal hernia. Femoral hernia and direct and indirect inguinal hernia are grouped together as groin hernia. Although femoral hernia is relatively rare, accounting for only 2-4% of all groin hernias, its importance is due to the high incidence of incarceration and strangulation^12^ ^,^ ^13^ ^,^ ^26^. Although femoral hernia is 8 to 10 times more common in females than males, indirect inguinal hernia is the most common hernia in both genders^1^ ^,^ ^6^ ^,^ ^18^ ^,^ ^23^ ^,^ ^24^. 

Because of its elevated tendency to complications, patients with femoral hernia should be operated upon electively as soon as possible^1^ ^,^ ^5^. Emergency operations due to incarceration strangulation, intestinal obstruction and other complications are associated with expressive morbidity and mortality^7^ ^,^ ^8^ ^,^ ^10^. A multitude of surgical operations have been employed to treat femoral hernias, including open inguinal or femoral access, laparoscopic procedures, use of mesh or plugs and closure of the femoral canal^3^ ^-^ ^5^ ^,^ ^14^ ^,^ ^15^ ^,^ ^17^. In the last decade, laparoscopic access became the preferable access to treat groin hernia for most surgeons^17^ ^,^ ^20^ ^,^ ^32^. In addition, to allow correction of associated groin hernias either in the same side or bilaterally in a single access, laparoscopic operation has several advantages over conventional open techniques, such as less pain, short recovery period, and better esthetic results^11^ ^,^ ^16^ ^,^ ^25^. Publications on laparoscopic treatment of femoral hernias are limited. 

This research has the objective to assess the outcome of femoral herniorrhaphy employing the totally extraperitoneal laparoscopic (TEP) access.

## METHODS

Of a total of 2,399 patients who underwent laparoscopic groin herniorrhaphy between August 1998 and February 2020 in the Department of Surgery at the Hospital Nossa Senhora das Graças, Curitiba, Brazil, 62 (2.6%) had femoral hernia. The electronic medical records and study protocols of all patients were retrospectively reviewed. The present study was approved by the Ethics Committee of the Clinical Hospital of the Federal University of Parana (Nº 4.320.933).

As recommended by the European Hernia Society, femoral hernia was defined as tissue protruding through a well-defined hernia ring below the iliopubic tract and medial to external iliac vein^32^. Clinical diagnosis of femoral hernia was established by presence of mass or bulging in the inguinal and/or femoral region. Ultrasonography (n=16), computed tomography (n=5), and magnetic resonance (n=2) were performed preoperatively to confirm the diagnosis.

In seven patients, diagnosis of femoral hernia was established only at laparoscopic inspection of the preperitoneal pelvic region of scheduled inguinal herniorrhaphy. Preoperatively, four of these patients were erroneously diagnosed as recurrent inguinal hernia, but only a femoral hernia was observed at operation. The preoperative diagnosis of inguinal hernia was not confirmed in these four patients. In the other three patients, an unsuspected femoral hernia was diagnosed at laparoscopic inspection in addition to the inguinal hernia recognized prior to the operation.

### Surgical procedure

Femoral herniorrhaphy was routinely started with totally extraperitoneal (TEP) repair, independent of the presence of previous abdominal operations or hernia recurrence. The procedure was previously described^9^. Briefly, carbon dioxide (CO_2_) was initially insufflated into the preperitoneal space immediately superior to the pubic symphysis (Space of Retzius) through a Veress needle inserted into the midline just above the pubis. Three trocars (a 10-mm infraumbilical, a 5-mm left-flank, and a 10-mm right-flank trocar) were introduced with no balloon dissector. A 15x15 cm polypropylene mesh was placed into the preperitoneal space. A 3 cm slit was made in the mesh to encircle either the round ligament or the spermatic cord. In most patients the mesh was secured on the posterior aspect of the abdominal wall by intra-abdominal pressure alone, with no fixation (sutureless). However, in patients with large hernia, tacks were employed to fix the mesh.

In patients in which it was impossible to complete the operation with the TEP procedure due to technical difficulties, the TAPP (transabdominal preperitoneal) procedure or open herniorrhaphy was employed. 

A single intravenous dose of cephazolin 2 g was given at anesthesia induction. Enoxaparin sodium 40 mg was also injected subcutaneously at anesthesia induction in patients >50 years of age, obesity (BMI >30 kg/m^2^), malignant tumor or presence of other risk conditions.

Immediately prior to wound incision for trocar insertion, all abdominal layers at the trocar sites were infiltrated with local anesthetic (bupivacaine hydrochloride 0.5%). The patients received a single intraoperative dose of intravenous parecoxib sodium 40 mg, tramadol hydrochloride 100 mg, and dipyrone 2 g for analgesia. A single dose of 4 mg of ondansetron was also administered intravenously prior to completion of the procedure to prevent postoperative nausea and vomiting. Liquid diet was started as soon as the patient was fully awakened and had no nausea and vomiting, usually 3-4 h after the operation. Normal diet was advanced as tolerated. No gastric tube or urinary catheter was used routinely.

The following data were obtained and analyzed: age, gender, history of prior groin herniorrhaphy, American Society of Anesthesiology score (ASA), operative findings, surgical technique, duration of operation, intra and postoperative complications, length of hospital stay, hospital readmission and hernia recurrence. Indications for conversion to either transabdominal preperitoneal (TAPP) repair or open herniorrhaphy were also analyzed. 

Patients were discharged on the same day of the operation with orientation to return to normal diet and activity as soon as tolerated. Lifting weight was limited to 10 kg in the first month after surgery. Patients returned for ambulatory follow-up on the 7^th^ day, and one and three months after operation. Follow-up was extended as needed in presence of clinical manifestations, complications, or hernia recurrence. Values are expressed as mean ± standard deviation.

## RESULTS

### Demographic and clinical characteristics

The mean age of the patients was of 58.9±15.9 years (22-92,Table 1). There were 55 (88.7%) females and 7 (11.3%) males, with a female to male ratio of 8:1 (Table 1). Most patients (n=53, 85.5%) had single hernia and the remaining (n=9, 14.5%) bilateral, making a total of 71 hernias operated.

Prior lower abdominal operations were recorded in 21 (33.9%) patients. The most common surgeries were gynecological procedures, including cesarean section (n=5, 8.1%), hysterectomy (n=3, 4.8%), and tubal ligation (n=3, 4.8%). Six patients (9.7%) had previous lower abdominal herniorrhaphies, including inguinal (n=4, 6.5%) and incisional (n=2, 3.2%). Four patients referred prior appendectomy (n=3) and colectomy (n=1). Four patients (6.5%) underwent concomitant operations in addition to the femoral hernia correction: umbilical herniorrhaphy in three and laparoscopic cholecystectomy in one.

Table 1 also displays the preoperative American Society of Anesthesiologists (ASA) score distribution. Most patients had score I (normal healthy patients, n=24, 38.7%) or score II (patient with mild systemic disease, n=31, 50.0%). Five patients (8.1%) had score III (patient with severe systemic disease that is not life-threatening) and two (3.2%) score IV (patient with severe systemic disease that is a constant threat to life).

**TABLE 1:** Patient demographic and clinical characteristics

Characteristics	n	%
Number of patients Number of hernias Age (years) Mean ± SD Range Gender Female Male Female to male ratio Hernia Side Right Left Bilateral Recurrent hernia Concomitant operations Prior abdominal Surgery ASA Score I II III IV	62 71 58.9 ± 15,9 24-92 55 7 28 25 9 1 4 21 24 31 5 2	88.7 11.3 45.2 40,3 14.5 2.2 6.5 33.9 38.7 50.0 8.1 3.2

ASA= American Society of Anesthesiology

### Operative aspects

The mean operative time was 37±12 min for the unilateral hernias and 59±16 min for the bilateral. Conversion to TAPP procedure was performed due to technical difficulties to dissect the preperitoneal space in four patients (6.5%) due to the presence of intense abdominal wall fibrosis in patients with previous abdominal operations. 

Open herniorrhaphy was needed in two patients (3.2%). One of these patients presented with spontaneous enterocutaneous fistula in the groin region. No history of trauma or intestinal obstruction was referred. Radiographic fistulography performed with injection of contrast media through the cutaneous orifice confirmed the diagnosis of enteric fistula communicating the groin skin with a distal small bowel loop. At the operation, performed through an incision in the inguinal region, a Richter hernia was recognized. A small portion of the circumference of the ileum was incarcerated and perforated into the femoral canal. The ileum segment involved was resected and an ileo-ileal hand-sewn anastomosis performed. Femoral hernia was treated with suture of the transversalis fascia to Cooper’s ligament and lacunar ligament (McVay technique). No mesh was employed due to fecal contamination of the femoral region.

Open hernia correction was also performed in another patient with incarcerated femoral hernia. An incidental perforation of the adjacent small bowel occurred during dissection of the hernia sac. The procedure was converted to an open operation with an incision in the inguinal region. After segmental enterectomy and primary reconstruction of the small bowel transit, the femoral hernia was repaired using the McVay technique with no mesh placement.

### Surgical complications

Intra- and postoperative complications occurred in four (6.5%) and five (8.1%) patients, respectively. There was no mortality. The complications are shown in Table 2.

**TABLE 2:** Intraoperative and postoperative complications

Intraoperative complications	n	%
Inferior epigastric vessel injury	2	3.2
Small bowel perforation	1	1.6
Skin burning	1	1,6
		
Postoperative Complications		
Urinary retention	1	1.6
Thrombophlebitis	1	1.6
Wound infection	1	1.6
Urinary infection	1	1.6
Pneumonia	1	1.6

Two patients (3.2%) had injury of the inferior epigastric vessel either at trocar insertion or during areolar tissue dissection of the preperitoneal space. Bleeding was easily controlled by clipping, with no further complication. The patient who had small bowel perforation during sac dissection of an incarcerated femoral hernia had an uneventful recovery after treatment with enterectomy and was discharged on the 4^th^ postoperative day with no further complication.

One patient had an accidental 3 cm by 3 cm sized partial-thickness skin burn in the left leg where the grounding pad of the electrosurgical device had been placed. The patient was treated conservatively with dressings with topical silver-sulfadiazine.

Urinary retention that required catheterization was recorded in one patient (1.6%). He had lower urinary tract infection two weeks later, which was treated with antibiotics.

Superficial wound infection at the umbilical trocar insertion site occurred in one patient (1.6%) and was treated with local dressing with povidone-iodine. No antibiotic was used. Thrombophlebitis and pneumonia were diagnosed in one patient each. These complications were treated conservatively.

### Hospital discharge and follow-up

Most patients (n=52, 84%) were discharged from the hospital on the same day of the operation. Ten (16%) stayed in the hospital from 1-7 days mainly due to refusal to be discharged (n=5, 8.1%), nauseas and vomiting (n=2, 3.2%), and urinary retention (n=1, 1.6%). The two (3.2%) who underwent small bowel resection stayed in the hospital for four and seven days. Two (3.2%) were readmitted to the hospital due to thrombophlebitis (n=1, 1.6%) and pneumonia (n=1, 1.6%). 

Sixty patients (96.8%) had a follow-up period of at least three months (5.36±2.53). Hernia recurrence was diagnosed in two (3.2%). The two recurrences occurred in the first three months.

## DISCUSSION

Femoral hernia is relatively rare and its incidence is only 2-4% of all groin hernias^12.13,26^. Femoral hernias occur in both genders but they are far more common in females than in males, in a ratio of about 10: 1^18^ ^,^ ^19^ ^,^ ^21^. Although femoral hernia may occur in any age, including infants under one year, its prevalence increases with age, with a mean age of about 60-70 years. The female to male ratio of 8: 1 and the mean age of 59 years observed in our study are similar to results of several other reports^18^.

Femoral hernia may be effectively treated with a multitude of open, laparoscopic, and robotic techniques^27^ ^-^ ^31^. The procedure selection depends on surgeons’ expertise and preference, patient- and hernia-related characteristics, costs, and local availability^1^ ^,^ ^2^. The international guidelines for management of groin hernias (HerniaSurge Group) indicates that laparoscopic repair has the advantage of diagnosing other groin hernias which may not have been recognized preoperatively^18^. Crawford et al.^11^ have diagnosed unsuspected femoral hernias in 27 of 253 patients (11%) who underwent laparoscopic groin repair. In addition, laparoscopic approach is associated with faster recovery time, lower chronic pain, and lower risk. 

We have employed the TEP approach to routinely correct femoral hernias for the last 25 years. According to our protocol, we always start with TEP access in all patients with femoral hernia, independent of the presence of previous abdominal operations, hernia recurrence, or incarcerated hernia. As compared to TAPP (Laparoscopic Transabdominal PrePeritoneal), TEP has the advantage of not entering the peritoneal cavity and therefore reducing abdominal viscera lesions, especially in patients with intra-abdominal adherence. However, some surgeons avoid or contraindicate the TEP and even the TAPP approach in patients with previous lower abdominal operations because of the difficulty to dissect the preperitoneal space and to prevent intra-abdominal organ injuries^22^ ^,^ ^33^ ^,^ ^34^.

Femoral hernia is associated with high incidence of complications, such as incarceration, strangulation, and necrosis of several organs and tissues, such as epiploon, small or large bowel, appendix, ovary, Fallopian tube, bladder, and Meckel’s diverticulum^22^ ^,^ ^27^ ^,^ ^31^ ^,^ ^33^. Because of the small neck of the hernia, obstruction and strangulation are frequent, often without apparent external signs of a hernia. Emergency operation with its elevated risks is needed in case of complications, regardless of the condition or age of the patient. Delay in recognizing incarceration and strangulation may cause devastating complications, such as bladder and intestinal perforation, abdominal wall and pelvic abscesses, Fournier’s gangrene, and urinary and intestinal fistulas to the skin, vagina, and scrotum^1^ ^,^ ^13^ ^,^ ^14^ ^,^ ^33^. In the presence of these complications, mortality may be expressive.

One of our patients presented with spontaneous enterocutaneous fistula in the groin region due to perforation of a strangulated small bowel loop in a femoral hernia. There was no history of trauma or intestinal obstruction. Richter’ hernia was confirmed by fistulography and operative findings. 

 Richter’s hernia is a very uncommon medical condition that is difficult to diagnose before complications are identified^14^. It is characterized by incarceration of a small portion of the circumference of the intestine wall within a hernia, which leads to ischemia, gangrene, and perforation of the bowel. Because the incarceration is limited to just a portion of the antimesenteric bowel wall, no intestinal obstruction occurs. It usually occurs in small hernia rings large enough to entrap the partial circumference of the bowel wall, but small enough to prevent penetration of an entire loop of the intestine^1^ ^,^ ^14^. That is the reason why the femoral canal with its firm margins is the main location of Richter’s hernia. Richter´s hernia is also observed in other hernia sites, such as inguinal region, Spigelian line, drain site and incision site. With the widespread use of laparoscopic procedures, laparoscopic port site has become a common site of Richter’s hernia^1^ ^,^ ^14^ ^,^ ^33^.

Similar to most authors, we opted to treat our patient with Richter´s hernia with resection of the bowel loop incarcerated with reconstitution of the intestinal transit in one-stage. The femoral hernia was treated with McVay technique with no mesh to avoid infection. However, some authors prefer one-stage repair with mesh in the absence of peritonitis, severe ileus and/or bowel necrosis^7^. These surgeons do not use mesh only in patients with serious infection or combined fistula or who need radical debridement. 

More recently, some surgeons have demonstrated the safety and feasibility of the laparoscopic approach to treat incarcerated, and even strangulated Richter femoral hernia, which was appropriately corrected with laparoscopic hernioplasty combined with intestinal resection assisted by laparoscopy^14^.

The surgeon must use the utmost caution in reducing the intestine in patients with incarcerated femoral hernia in order to avoid intestinal perforation. We had a perforation of the small bowel during femoral hernia reduction from the femoral ring with its firm edges. The patient had an uneventful recovery following conversion to open surgery with enterectomy and hernia repair with no mesh. Inadvertent lesions of the small and large bowel, bladder and other organs have also been reported by others.

Femoral hernia recurrence is low. Four of our female patients were subjected to previous inguinal herniorrhaphies in other institutions. Although some authors have suggested that prior inguinal hernia repair might be a risk factor for femoral hernia development, most believe that this association is inaccurate^29^. The occurrence of femoral hernia after inguinal herniorrhaphy is possibly due to unrecognized femoral hernia during the first operation and not a new hernia. Femoral hernias may be overlooked at open inguinal herniorrhaphies despite digital exploration of the femoral canal^16^. 

In a systematic review of recurrence after inguinal hernia repair in females, Schmidt et al.^29^ reported that 203 of 496 patients (40.9%) with recurrent hernia had femoral hernia after open procedure compared with no recurrence after laparoscopic repair. These findings suggested that the high development of femoral hernia after inguinal herniorrhaphy was due to non-identification of femoral hernia in the initial operation because of its deep location and small size. On the contrary, laparoscopic access allows easy evaluation of the femoral region, including a possible identification of femoral hernia, justifying the absence of recurrence after laparoscopic procedure, but frequent occurrence after open herniorrhaphy. Several studies have documented the diagnosis of unsuspected femoral hernias in patients subjected to inguinal hernia correction by laparoscopy^11^ ^,^ ^16^. Crawford et al.^11^ have diagnosed unsuspected femoral hernias in 27 of 253 patients (11%) who underwent laparoscopic groin repair.

The major limitation of our study is the retrospective evaluation of the data of our patients and the short follow-up. This is minimized because all surgical procedures were coordinated and supervised by only two surgeons and the data were retrieved from electronic medical records and study protocols. Another limitation refers to our limited follow-up time. Although our mean follow-up was not long enough to exclude late recurrences, most groin hernia recurrences occur in the first few months following herniorrhaphy. The major strength of our study is the large sample size of patients with femoral hernia treated with TEP procedure in a single institution. We routinely started with the TEP approach in all patients with femoral hernias, independent of the presence of previous abdominal operations or hernia recurrence.

## CONCLUSION

Femoral hernia is uncommon, and it may be associated with life threatening complications. Most femoral hernias may be successfully treated with totally extraperitoneal laparoscopic technique, with low conversion and complication rates.
